# Comparative study of mesenchymal stem cells from C57BL/10 and mdx mice

**DOI:** 10.1186/1471-2121-9-24

**Published:** 2008-05-19

**Authors:** Yong Li, Cheng Zhang, Fu Xiong, Mei-juan Yu, Fu-lin Peng, Yan-chang Shang, Cui-ping Zhao, Yong-feng Xu, Zheng-shan Liu, Chang Zhou, Jin-lang Wu

**Affiliations:** 1 Department of Neurology, the First Affiliated Hospital, Sun Yat-sen University, Guangzhou, Guangdong, ProC; 2 Stem Cells and Tissue Engineering Research Center, Sun Yat-Sen University, Guangzhou, Guangdong, ProC; 3 Department of electron microscope, Sun Yat-Sen University, Guangzhou, Guangdong, ProC; 4 Department of Neurology, Second Affiliated Hospital, College of Medicine, Zhejiang University, Hangzhou, ProC

## Abstract

**Background:**

Human mesenchymal stem cells (MSCs) have been studied and applied extensively because of their ability to self-renew and differentiate into various cell types. Since most human diseases models are murine, mouse MSCs should have been studied in detail. The mdx mouse – a Duchenne muscular dystrophy model – was produced by introducing a point mutation in the dystrophin gene. To understand the role of dystrophin in MSCs, we compared MSCs from mdx and C57BL/10 mice, focusing particularly on the aspects of light and electron microscopic morphology, immunophenotyping, and differentiation potential.

**Results:**

Our study showed that at passage 10, mdx-MSCs exhibited increased heterochromatin, larger vacuoles, and more lysosomes under electron microscopy compared to C57BL/10-MSCs. C57BL/10-MSCs formed a few myotubes, while mdx-MSCs did not at the same passages. By passage 21, mdx-MSCs but not C57BL/10-MSCs had gradually lost their proliferative ability. In addition, a significant difference in the expression of CD34, not Sca-1 and CD11b, was observed between the MSCs from the 2 mice.

**Conclusion:**

Our current study reveals that the MSCs from the 2 mice, namely, C57BL/10 and mdx, exhibit differences in proliferative and myogenic abilities. The results suggest that the changes in mouse MSC behavior may be influenced by lack of dystrophin protein in mdx mouse.

## Background

Similar to hematopoietic stem cells, mesenchymal stem cells (MSCs) are a type of stem cells derived from the bone marrow (BM). They were identified by Cohnheim in 1867 and were described by Friedenstein and colleagues [1]. MSCs exhibit remarkable plasticity and harbor potential for use in therapeutic applications, such as fibrosis [2], cardiovasculogenesis treatments [3], arteriogenesis [4], and immunosuppression [5]; they can also be used in tissue engineering [6] and the correction of genetic disorders [7].

As a research tool, MSCs offer the advantages of easy manageability, versatility, and the ability to proliferate for prolonged periods without undergoing transformation [8]. The pleiotropic immune-related properties of MSCs (low immunogenicity and lack of alloreactivity) have potential for achieving haematopoietic stem ell transplantation (HSCT) with a low incidence of GVHD (graft-versus-host disease) [9,10]. In addition, the low cost of maintaining mice and the detailed knowledge of mouse genetics [11] favor the utilization of murine MSCs (mMSCs) for extensive studies in the field of adult stem cell research.

Duchenne muscular dystrophy (DMD) is a common recessive X-linked, monogenic muscular disorder with an incidence of 1 in 3,500 male births. The primary genetic defect leads to the near absence of dystrophin, resulting in muscle damage and wasting. DMD boys experience progressive muscle wasting and weakness that becomes apparent by 3–5 years of age and are wheelchair-bound by 12 years of age [12]. The research model that is most commonly employed for DMD is the mdx mouse. This mouse has a point mutation in the 5'-end of its dystrophin gene (exon 23) that creates a stop codon and an unstable truncated protein and results in the complete loss of the full-length dystrophin protein [13].

Many researchers have employed MSCs derived from different sources to enhance dystrophin expression and ameliorate symptoms of mdx mice [14-18]. However, little is known about the behavioral characteristics and functions of mdx MSCs. Further, there is not much information about the structural and ultrastructural differences between mutant mouse mesenchymal stem cells (mMSCs) (mdx mouse) and normal mMSCs (C57BL). In this study, we investigated the differences in the morphological changes and colony-forming efficiency between adult C57BL/10 and mdx MSCs by light and electron microscopy; further, we investigated the behavioral differences between these 2 types of MSCs by flow cytometry. Since the genetic  background of both the C57BL/10 and mdx mice is very  similar [19], the absence of the dystrophin protein may result in a change in the features of the mdx MSCs.

## Methods

### Isolation of stem cells and cell cultures

C57BL/10 adult mice were purchased from NICPBP(National Institute for the Control of Pharmaceutical and Biological Products)(Beijing, China), mdx (C57BL/10ScSnJ) adult mice were purchased from The Jackson Laboratory (Me, USA).

The local ethics committee approved the animal experimentation protocols and all animal experiments were performed according to Sun-Yet university guidelines for animal care. MSCs were harvested from femur and tibia bone marrow of C57BL/10 and mdx adult mice (6–8 weeks). The mice were housed in identical cages and allowed access to water and a standard rodent diet shu liang. All chemicals were purchased from Sigma (St. Louis, MO) unless otherwise noted.

In brief, both C57BL/10 and mdx mouse were killed by cervical dislocation and bone marrow was flushed out of tibias and femurs with Dulbecco's modified Eagle's medium (DMEM) containing 10% fetal bovine serum (FBS) and 2 mM L-glutamine. After washing by centrifugation at 400×g for 10 min and counting of viable trypan blue-excluding cells in a Neubauer chamber, the cells were resuspended with DMEM to a final concentration of 5×10^6 viable cells per millilitre. To initiate an mMSC culture, cells were plated in six-well tissue culture dishes (CELLSTAR, Greiner, German), at 2×10^6 cells/cm2. The culture was kept in a humidified 5% CO2 incubator at 37°C for 72 h, non-adherent cells were removed by changing the medium. When the adherent cells reached confluence, covering 70%~80% of culture dish, the cells were washed once with phosphate-buffered saline (PBS). A 0.25% trypsin solution containing 0.01% ethylenediaminetetraacetic acid (EDTA) incubated on monolayer at 37°C for 10 min. After detachment of the adherent cells with a pipette, cells were resuspended in medium to a final volume of 7 ml, and the resulting suspension was split into two new wells. Subsequent passages were performed similarly, except that incubation was for 5 min at room temperature (RT) and split ratios were 1:2. After passage 4, the incubation with trypsin was less than 2 min. and the split rate was set to 1:3. The culture medium was changed every 3–4 days.

### Light and electron microscopy

The mMSC cultures were routinely observed on an inverted research microscope (Olympus, IX71, Japan). Morphological analysis was carried out at P9 (53–63 days of culture), P19 (88–98 days of culture), P25 (109–119 days of culture). Photomicrographs were taken with a charge coupled device (CCD) camera (COOLSNAP-Procf, Sony, Japan) coupled to an inverted microscope, using Image-pro Express software.

For electron microscopy, the medium was discarded from the flask containing mMSCs, which were fixed in 1% paraformaldehyde, 1.25% glutaraldehyde in 0.1 M PBS (pH 7.2) for 20 min. The cells were scraped from flask with cell scraper, centrifuged at 800 g for 10 min and formed a cell pellet in the bottom of centrifuge tube. The pellet was then washed three times in 0.1 M PBS (pH 7.2), post-fixed in 2% osmium tetroxide, dehydrated and embedded in Epon812, which consists of dodecenylsuccinic anhydride (23.5%), methyl nadic anhydride (72.5%), supplemented with 4% of the accelerator DMP-30 (PELCO). The plasticizer dibutyl phthalate was added at 0.5%. Thin sections (50 nm) were cut on a LKB-I Ultracut UCT, stained with uranyl acetate and lead citrate, and examined in a Hitachi-600 transmission electron microscope (Japan). 65 mdx-MSCs and 73 C57BL/10-MSCs were counted to assess morphology in transmission electron microscope (TEM).

### Colony forming units-fibroblast (CFU-F) assay

The CFU-F assay was performed using a modification of a described protocol [20]. Cells cultured were resuspended in the above medium to a concentration of 10 viable cells/ml. Ten millilitre of this cell suspension was plated in a 10 cm Petri dish (1.7 cells/cm2).

The medium was changed 2 times per week. On the 13th day, cultures were fixed and stained with Giemsa. Fibroblastic colonies with more than 50 cells were counted under an inverted microscope. Each mMSCs were performed in triplicate in three C57BL/10 or mdx mice.

### Flow cytometry

Cells were trypsinized, collected and incubated for 30 min at 4°C with phycoerythrin (PE)- or fluorescein isothiocyanate (FITC)-conjugated antibodies against murine Sca-1 (stem cell antigen 1), CD11b, CD34 and IgG (Pharmingen, USA). Excess antibody was removed by washing. Detection of PE and FITC labeling was accomplished on a FACScalibur cytometer (Becton Dickinson, San Jose, USA) using CELLQuest software.

### Differentiation of mMSCs

For osteogenesis, the cultures were incubated in DMEM supplemented with 10% FBS, 2 mM L-glutamine, 10 mM β-glycerol phosphate, 1 × 10^-8 ^M dexamethasone, and 5 mg/L ascorbate 2-phosphate. The medium was changed 2 times per week for 3 weeks. The cells were fixed with 10% formalin for 20 minutes at RT and stained with Alizarin Red, pH 4.1 for 20 minutes at RT.

For adipogenesis, the cultures were incubated in DMEM supplemented with 10% FBS, 2 mM L-glutamine, 5 μg/mL insulin, 1 × 10^-8 ^M dexamethasone. The medium was changed 2 times per week for 3 weeks. The cells were fixed with 10% formalin for 20 minutes at RT and stained with 0.5% Oil Red O in methanol for 20 minutes at RT.

For myogenesis, 5×10^3 cells/well were incubated in DMEM supplemented with 10% FBS, 2 mM L-glutamine, treated for 24 hours with 10 μmol/L 5-azacytidine in 10% DMEM. 18 pieces of 6-well plates of C57BL/10-MSCs and mdx-MSCs at passage 5, 10 and 13 were incubated overnight in a 37°C incubator with a humidified atmosphere of 5% CO2. The following day, the supernatant was removed and replaced with 5% horse serum in DMEM solution for 4 weeks. The medium was changed 2 times per week. For the determination of MyHC (Myosin Heavy Chain), and dystrophin, the cells were fixed with 4% paraformaldehyde for 20 minutes at RT, followed by incubation with anti-MyHC-antibody (Santa Cruz Biotechnology, Santa Cruz, CA) and anti-dystrophin (Santa Cruz Biotechnology, Santa Cruz, CA). Secondary antibodies were coupled with Cy3 (red) and used according to the manufacturer's instructions. Nuclei were visualized using a 30 μM 4',6'-diamidino-2-phenylindole (blue).

Images were processed by an inverted research microscope (Olympus, IX71, Japan). Digital images were analyzed using Image-pro Express software

### RNA purification and gene expression analysis by RT-PCR after induction

After both MSCs were induced for three weeks, total cellular RNA was isolated using Trizol (Gibco-BRL, Life Technologies, MD). Random hexamer-primed reverse transcription (MBI Fermentas Inc., Burlington, ON) was performed on aliquots (1 ug) of total RNA as a template and used the resultant cDNA for PCR amplification. Primers for osteocalcin (OCN), lipoprotein lipase (LPL), and glyderaldehyde-3-phosphate dehydrogenase (GAPDH) were synthesized based on the reported sequences. OCN (199 bp): forward: 5'-TCTGACAAAGCCTTCATGTCC-3', reverse: 5'-AAATAGTGATACCGTAGATGCG-3'; LPL (557 bp): forward: 5'-ACTCATCTCCGCCATGCC-3', reverse: 5'-CCAGCTTTCTCCTAGCAAGG-3'; and GAPDH (203 bp): forward: 5'-TGACCACAGTCCATGCCATC-3', reverse: 5'-GACGGACACATTGGGGGTAG-3'. Reaction mixtures (30 ul) contained 1 ul cDNA, 30 pmol of each primer, 3 ul of 200 uM dNTP, and 1U Taq-DNA polymerase (MBI Fermentas Inc., Burlington, ON). Amplification conditions were as follows: 25 cycles of 94°C for 30 s; 55°C for 60 s; and 72°C for 1 min, followed by a 72°C incubation for 10 min. The PCR products were detected by 1.2% agarose gel electrophoresis and photographed.

### Statistical analysis

Data are presented as mean ± standard deviation. A two-sided, paired t test was used to analyze the flow cytometry and the isolation efficacy of CFU-F. Differences were considered significant at p < 0.05. The SPSS software package (version 12.0) was used for the statistical tests.

## Results

### Morphology under light microscopy

BMs were isolated from 12 mice (C57BL/10 and mdx). MSCs were isolated successfully from 8 mice – 5 C57BL/10 and 3 mdx mice. The characteristics of 3 C57BL/10 and 3 mdx MSCs were examined.

**Figure 1:**
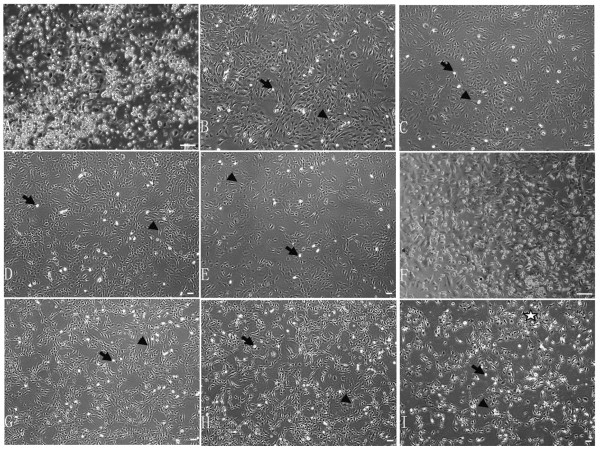
Phase contrast images of C57BL/10 MSCs (A-E) and mdx MSCs (F-I). Passage 0 (A, F), passage 9 (B, G), passage 19 (C, H), passage 25 (D, I), and passage 40 (E). Round cells (A-I: thin black arrows). Spindle-shaped cells (A-I: arrowheads). Cells in Fig. G were more spindle-shaped than those in Fig. B. Cells in Fig. H to Fig. C showed the same result. Mesh and net formation was observed between the cells in Fig. I (pentagram). Scale bar, 200 μm.

The C57BL/10 and mdx mMSCs were generally observed to be morphologically highly heterogeneous before passage 5 and comprised round, spindle-shaped, and flattened cells (Fig. 1A, F). After passage 5, the morphology of most cells was spindle-shaped, and few round cells were observed (Fig. 1B–E, 1G–H). The population of spindle-shaped mdx MSCs expanded gradually over time. These cells ceased proliferating (Fig. 1I) before passage 25. However, the morphology of C57BL/10 MSCs was not altered and these cells continued to proliferate even after passage 40 (Fig. 1E).

### Morphology under TEM

**Figure 2:**
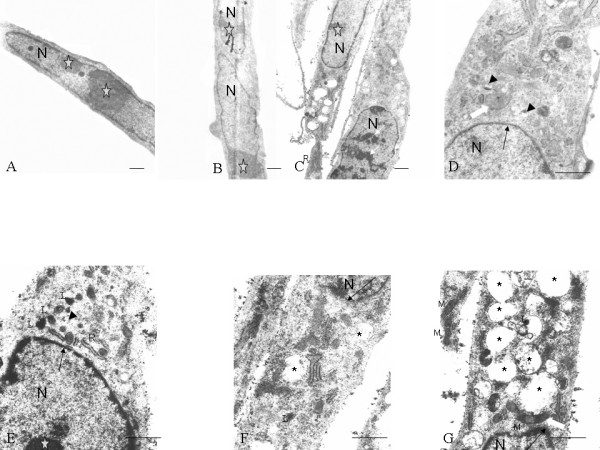
Electron microscopic images of both C57BL/10 MSCs (A, B, D) and mdx MSCs (C, E, F, G). All cells showed similar morphological features: an elliptical nucleus (N), usually with multiple nucleoli (pentagrams); various mitochondrial profiles; and small vacuoles (D, E: arrowheads). Big vacuoles (F, G: asterisks) and lysosomes (E-G: L) were observed in the cytoplasm of mdx MSCs, and heterochromatin was observed in the nucleus of these cells (C: thin white arrows). Chromatin formed a thin and dense layer inside the perinuclear cisternae (D-G: thin black arrows). Mitochondria (M) with both round and elongated profiles (D, G: thick white arrows) were observed. Scale bar, 1 μm.

At low magnification (Fig. 2A–C), the C57BL/10 and mdx mMSCs at passage 10 appeared similar. They had a large elliptical nucleus, with one or more nuclei located generally near the perinuclear cisternae (Fig. 2A–C). The chromatin was dispersed except for a thin dense layer located immediately inside the perinuclear membrane (Fig. 2A–G).

Ultrastructural observations indicated intact organelle structure and ribosome-rich cytoplasm (Fig. 2D, G). Rough endoplasmic reticulum and mitochondria were detected in both the inner and peripheral endoplasmic zones (Fig. 2C, G). The endoplasmic reticulum was often observed to be expanded (Fig. 2D, E), giving the cytoplasm a small vacuolated appearance.

The morphological changes in 65 mdx MSCs at passage 10 and 13 under TEM were as follows: 3 (4.6%) cells exhibited heterochromatin increase (Fig. 2C); 10 (15.4%), large vacuoles (Fig. 2F, G); and 5 (7.7%), lysosomes (Fig. 2E–G). No such changes were observed in the C57BL/10 MSCs at the same passage level.

**Figure 5:**
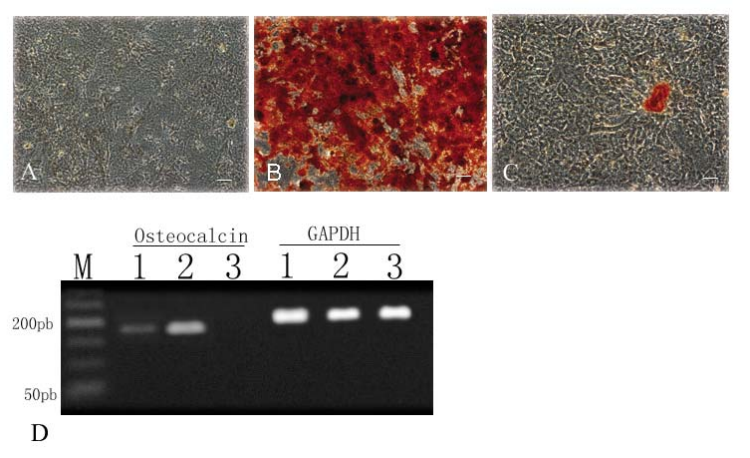
Osteogenesis of both mMSCs. MSCs (3 C57BL/10 MSCs and 3 mdx MSCs) were induced in an osteogenic medium, and osteoblast differentiation was indicated by alizarin red staining. Negative control (A), C57BL/10 MSCs (B), and mdx-MSCs (C). The osteocalcin gene was detected in induced osteoblasts by RT-PCR. Lane 1-mdx MSCs, Lane 2-C57BL/10 MSCs, and Lane 3-negative control (D). Lane M, marker; GAPDH, glyderaldehyde-3-phosphate dehydrogenase; OCN, osteocalcin. Scale bar, 100 μm.

### The growth kinetics and population doubling time of both MSCs

**Figure 3:**
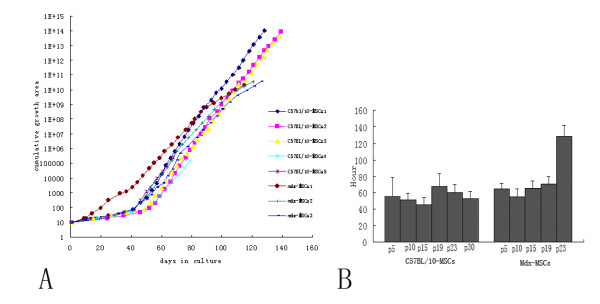
Growth kinetics and population doubling time of both MSCs. The value 1 was substituted for the growth area (GA) occupied by a primary mMSC culture, which corresponded to 9.03 cm^2^, to simplify the calculation. When the second passage took place, the split ratio at passage 1 (1:3) was multiplied by this value, meaning that at the end of passage 1, the cumulative GA was 3 (i.e., 3 times the growth area occupied by the primary culture). At the end of the second passage, the split ratio at passage 2 (1:2) was multiplied by the cumulative GA at passage 1 (3), resulting in a cumulative GA value of 6 at passage 2. This procedure was repeated for each passage, providing a theoretical growth curve that is directly proportional to the cell number. C57Bl/10: normal, male, 6–8 weeks old; mdx: male, 6–8 weeks old (A). A known number of MSC types from different passages were cultured. The total cover area of cells in the flask was determined at different time points to obtain the doubling time. The values are expressed as means ± SD of 3 independent measurements (B).

Under the conditions defined in this study, cell confluence was usually reached between 10 and 14 d. The interval between passages varied greatly until passage 5; thereafter, a passage interval of approximately 4 d was set. Continuous cell growth was observed in all cultures (Fig. 3A). In 5 C57BL/10 and 3 mdx MSCs, an increasing growth rate was observed that stabilized after 45 d in culture; however, in some C57BL/10 MSCs, a constant growth rate was observed till passage 40. The growth rate of mdx MSCs appeared to decrease after passage 21

The doubling time of C57BL/10 MSCs in culture was analyzed at multiple time points over extended culture periods. In C57BL/10 MSCs, no significant changes were observed between passages with regard to the time required for population doubling (45 h–68 h). However, the doubling time of mdx MSCs increased as the number of passages increased (Fig. 3B). The mdx MSCs at passage 30 could not be analyzed as the doubling time became so protracted that cells failed to reach this passage.

### CFU-F assay

The ability of C57BL/10 and mdx MSCs to form fibroblastic colonies was evaluated using a CFU-F assay. The number of CFU-F/100 cells obtained at passage 15 is described in Additional tab. 1. Although the number of CFU-F formed per 100 cells of C57BL/10 MSCs was higher than that formed per 100 cells of mdx MSCs, the difference was not significant.

### Flow cytometry

**Figure 4:**
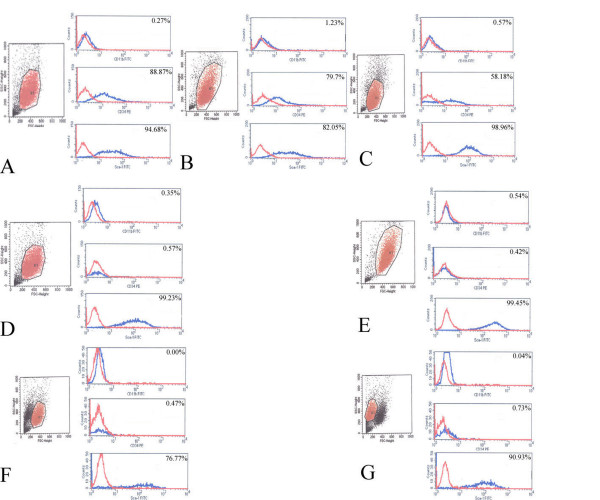
Immunophenotypic profile of both C57/BL10 and mdx MSCs. Characteristic homogeneous FSC-H × SSC-H plot exhibited by 5th passage C57BL/10 MSCs (A), 5th passage mdx MSCs (D), 9th passage C57BL/10 MSCs (B), 9th passage mdx MSCs (E), 19th passage C57BL/10 MSCs (C), and 19th passage mdx MSCs (F, G). Both types of mMSCs were incubated with antibodies against CD34, CD11b, and Sca-1 and assayed by FACS. Each antibody was tested individually, and representative plots from 3 samples from each strain are shown. Blue plot lines: negative control; red plot lines: MSCs.

Flow cytometric analyses revealed changes in the morphological homogeneity of 3 C57BL/10 and 3 mdx MSCs at passage 5, 9, and 19. A typical forward scatter (FSC-H) × side scatter (SSC-H) histogram (Fig. 4A–C) of C57BL/10 MSCs exhibited no distinct changes between the cells at passages 5, 9, and 19. However, a similar histogram of mdx MSCs indicated distinct changes in the morphology of these cells. The mdx MSCs at passage 5 and 9 appeared homogeneous (Fig. 4D, E) and those at passage 19 exhibited 2 distinct populations (Fig. 4F, G).

The surface expression of molecular markers in mMSCs (3 C57BL/10 and 3 mdx) at passages 5, 9, and 19 was evaluated (Fig. 4D–G). Both sets of cells were positive for Sca-1 and negative for CD11b, indicating that the mMSC populations were free of terminally differentiated hematopoietic cells. The expression of CD34 differed between C57BL/10 MSCs at different passage levels, whereas CD34 expression in mdx MSCs was always negative. There was a significant difference in the expression of CD34 between the 2 types of mMSCs, but no significant difference was observed between these cell types with regard to the expression of CD11b or Sca-1 (Additional tab. 1).

### Differentiation assays

**Figure 6:**
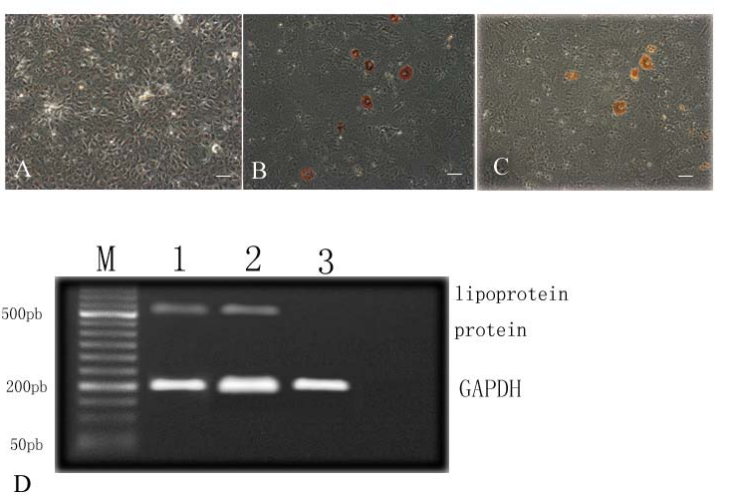
Adipogenesis of both mMSCs. MSCs (3 C57BL/10 MSCs and 3 mdx MSCs) were induced in an adipogenic medium. Adipogenic differentiation was indicated by oil red O staining. Negative control (A), C57BL/10 MSCs (B), and mdx MSCs (C). The expression of lipoprotein lipase was detected in induced adipocytes by RT-PCR. Lane 1-mdx MSCs, Lane 2-C57BL/10 MSCs, and Lane 3-negative control (D). Lane M, marker; GAPDH, glyderaldehyde-3-phosphate dehydrogenase. Scale bar, 100 μm.

When cultured MSCs (3 C57BL/10 and 3 mdx) were exposed to an osteoblast-induction medium, they formed aggregates, and calcium deposits appeared after 3 weeks. Alizarin red staining for calcium salt was performed. RT-PCR analysis also showed that another osteogenic-specific gene, namely, osteocalcin, was expressed in the cells after osteogenic induction (Fig. 5). The ability to differentiate into adipocytes was similar among MSCs derived from both mice. Using RT-PCR, we also showed that these cells expressed lipoprotein lipase, which is an adipocyte-associated gene (Fig. 6).

**Figure 7:**
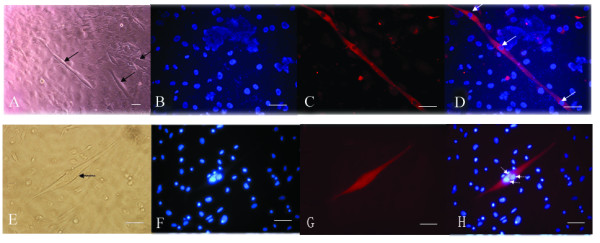
Myogenesis of C57BL/10 MSCs. Myotube formation in C57BL/10 MSCs at passage 13 (black arrows) (A, E), DAPI staining(B, F), MyHC (C) and dystrophin staining of a myotube (G), overlay of the MyHC (D) and dystrophin staining (H) (red fluorescence), DAPI staining (blue), and nucleoli (white arrows). Scale bar, 100 μm.

Of the C57BL/10-MSCs in 54 wells including 9 negative controls, MSCs at passages 5, 10, and 13 could form myotubes (Fig. 7) in 9 wells (9/45); however, mdx MSCs at the same passage levels did not, with most of the cells remaining flat after myogenic induction.

Both C57/BL10 MSCs and mdx MSCs were repeatedly differentiated individually 3 times and the similar results were found.

## Discussion

In both primary and passaged cultures of mMSCs, the unwanted growth of nonmesenchymal cells is observed. Therefore, the isolation of MSCs from mice is far more difficult than that from other species [21]. To resolve this problem, we used an isolation technique that is considered to be specific for separating mMSCs [22-26]. Differentiation assays showed that under appropriate culture conditions, mMSCs of C57BL/10 and mdx origin could differentiate into the adipocyte phenotype. This property is presently considered to be a critical requirement in identifying a putative MSC population [27,28]. However in this study, with regard to the osteogenesis ability of these mMSCs, the results of RT-PCR and alizarin red staining indicated that the osteogenesis ability of C57BL/10 MSCs was much greater than that of mdx MSCs (Fig. 5). Further research is necessary to clarify this finding.

Immunophenotyping of both types of mMSCs at passages 5, 9, and 19 showed that CD11b, which is a marker for granulocytes, monocytes, and natural killer cells, was absent in mMSCs derived from both mice. In our culture system, there was no need to sort CD11b-positive cells from the medium [29,30]. In addition, both mMSCs at different passage levels exhibited strong expression of Sca-1.

CD34 is a very interesting stem cell marker. It is expressed on the surface of HSCs, satellite cells, and endothelial progenitors. Its pattern and level of expression in muscle stem cells change as these cells differentiate into myotubes [31]. It is not expressed on the surface of the MSCs of most species [11,32]. However, some discrepancies were observed between the findings of studies on CD34 expression on the surface of mMSCs [33-36]. In our study, significant differences were observed between the 2 types of mMSCs with regard to the expression of CD34 (p < 0.05). This may result from differences in the niches of C57BL/10 and mdx mMSCs. It can also be attributed to the different behaviors of the 2 types of mMSCs. The mdx MSCs lost their proliferative capability gradually at passage 21 and formed a mesh-like network (Fig. 1I), and most cells decreased in size. These cells comprised a small population (Fig. 4F–G) at passage 19 and showed increased heterochromatin (Fig. 2C). Human MSCs and other cells show different morphological changes when they lose their proliferative ability, and senescent cells are larger and flatter than cells at other growth stages [37]. Compared to the changes observed in mdx MSCs, C57BL/10 MSCs showed persistent proliferative capability and no morphological changes over a period of 5 months. Meirelles et al [23] showed that C57BL/10 cells maintained homogeneous characteristics over a period of 8 months [23]. The correlation between proliferative ability and CD34 expression suggests that CD34+ cells may divide many more times and proliferate to a greater extent than CD34- cells [38,39]. In addition, although the colony-forming efficiency of the 2 types of mMSCs in this study was not significantly different, the colony-forming efficiency of CD34+ MSCs was greater than that of CD34- cells (Additional tab. 1). We found that C57BL/10 MSCs (CD34+ MSCs) could form myotubes (Fig. 7A, E) under suitable conditions [40], whereas mdx MSCs (CD34- MSCs) did not do so under comparable conditions. This also may be associated with differences between the 2 types of MSCs with regard to the expression of CD34 and imply that CD34+ MSCs may exert a greater role in myogenesis than CD34- MSCs [31,41,42]. This finding also suggested that CD34+ MSCs in the BM are one of the candidate cells involved in the formation of both myoblasts and myofibers. [43].

Another explanation for the differences between the 2 types of mMSCs with regard to myogenesis is that the dystrophin protein may play a role in myotube maintenance. The lack of dystrophin can lead to abnormal calcium homeostasis [44] and elevated calpain proteolysis [45] in the myotubes, resulting in a decrease in myotube formation.

There are only 5 published studies on the ultratructure of MSCs [28,46-49]. These focus on the changes in normal MSCs obtained after trypsinization. In our study, cells were scraped from flasks; these cells might better reflect the original cell state than cells treated with trypsin.

Two studies [48,49] showed that MSCs are "frequently binucleate," but according to a study by Raimondo et al [28], the 2 nuclei observed in MSCs can be attributed to the irregular shape of the nucleus. Our findings suggested that some binucleate mMSCs indeed existed (Fig. 2B). Three observations supported this: first, the nuclei in the MSCs were longer than those observed in other cells; second, each nucleus had its own nucleolus; and third, the space between the 2 nuclei was large.

Three studies [28,48,49] have reported the occurrence of vacuoles in the cytoplasm of MSCs. According to Raimondo et al [28], in most MSCs, vacuoles are formed as a result of the dilatation of the endoplasmic reticulum and Golgi apparatus. When the vacuoles in the C57BL/10 and mdx mMSCs were compared, the existence of 2 types of vacuoles was noted. The small vacuoles observed in both mMSCs had arisen from the dilatation of the endoplasmic reticulum and Golgi apparatus [28]. Large vacuoles were mainly observed in mdx MSCs in addition to lysosomes. They were formed as a result of cell degeneration, which could be one of the reasons for the loss in the proliferative ability of mdx MSCs.

In conclusion, our data showed that C57BL/10 MSCs (CD34+ MSCs) exhibited stronger proliferative capability and myogenic potential than mdx MSCs (CD34- MSCs). dystrophin protein would play main role in the changes of mouse MSC behavior.

Future research should be directed in 2 ways. First, the relationship between dystrophin and myotube characteristics such as myotube maintenance and myotube contraction should be determined; second, the proliferation and in vivo delivery of CD34+ MSCs should be investigated to clarify their potential application as therapeutic agents in mdx mice.
